# Effects of Copper on the System

**Published:** 1870-02

**Authors:** 


Effects of Copper on the System.—In a paper read before the Clinical Society of London, Dr. Clapton gave some interesting information concerning the effects of copper upon the system of workers in that metal. The presence of "distinctly marked green stains on the teeth, close to the gums, bluish-green perspiration, hair of a greenish hue, in old workmen, and green discharge from old ulcers," were described and exemplified by the presentation of patients, and specimens. These phenomena were shown to be probably the results of absorption, assimilation, and elimination of the metal. It is a singular fact that workers in copper are not only free from any specific poisoning beyond a tendency to muscular debility, but that they escape cholera and choleraic diarrhœa, even in localities where those diseases are epidemic.—*N. Y. Medical Gazette.*

---

A man in Tonganoxie, Kansas, thus encourages the editor of his favorite journal: "Continue to pour red-hot thunderbolts right into the teeth of the leeches and sharks that are sucking the life-blood from the people." Filling a bloodsucker's tooth with a red-hot thunderbolt must be a neat job in dentistry."

